# Thermal effects of headgear: state-of-the-art and way forward

**DOI:** 10.1186/2046-7648-4-S1-A71

**Published:** 2015-09-14

**Authors:** Cornelis C Bogerd, Jean-Marie Aerts, Simon Annaheim, Peter Bröde, Guido de Bruyne, Andreas D Flouris, Kalev Kuklane, Tiago Sotto Mayor, René M Rossi, TU1101 WG4 Action COST

**Affiliations:** 1 CBRN Protection, TNO, the Netherlands; 2 Division Measure, Model & Manage Bioresponses, KU Leuven, Belgium; 3 Laboratory for Protection and Physiology, Empa, Switzerland; 4 Leibniz Research Centre for Working Environment and Human Factors (IfADo), Dortmund, Germany; 5 Product Development, Faculty of Design Sciences, University of Antwerp, Belgium; 6 FAME Laboratory, University of Thessaly, Greece; 7 Department of Design Sciences, Lund University, Sweden; 8 HOPE-Helmet OPtimization in Europe, EU COST Action TU1101 Working Group 4 (http://www.bicycle-helmets.eu

## Introduction

Headgear is widely used in both work and leisure. Much research attention has been spent on optimizing impact properties of helmets [1], [2]. However, thermal comfort of headgear is suboptimal in neutral and warm environments. In fact, thermal discomfort is often given as a reason to not wear protective headgear [3], [4]. Enhanced thermal comfort of headgear is likely to improve the willingness to wear protective headgear, and motivated an increasing number of studies, of which most were published in the last decade. The available body of literature allows for a valuable first review on the thermal effects of headgear.

## Methods

The literature on thermal effects of headgear was reviewed for the purpose of providing a sound basis for improving helmet design, and for effective future studies.

## Results

Four topics will be addressed: (i) the effect on thermal physiology, health and performance, (ii) heat and mass transfer, (iii) methods for studying thermal effects of headgear, (iv) design considerations (Bogerd et al., 2015). Several topics will be detailed by other contributions to this conference from COST Action TU1101, which enhances the accessibility of the subject on ergonomics of headgear for the audience of this conference.
